# Health Care Utilization and Costs of Patients With Prostate Cancer in China Based on National Health Insurance Database From 2015 to 2017

**DOI:** 10.3389/fphar.2020.00719

**Published:** 2020-06-10

**Authors:** Lin Bai, Haishaerjiang Wushouer, Cong Huang, Zhenhuan Luo, Xiaodong Guan, Luwen Shi

**Affiliations:** ^1^Department of Pharmacy Administration and Clinical Pharmacy, School of Pharmaceutical Sciences, Peking University, Beijing, China; ^2^Center for Strategic Studies, Chinese Academy of Engineering, Beijing, China; ^3^School of Medicine, Tsinghua University, Beijing, China; ^4^International Research Center for Medicinal Administration, Peking University, Beijing, China

**Keywords:** China, prostate cancer, health care utilization, direct medical costs, burden of disease

## Abstract

**Background:**

In terms of medical costs, prostate cancer is on the increase as one of the most costly cancers, posing a tremendous economic burden, but evidence on the health care utilization and medical expenditure of prostate cancer has been absent in China.

**Objective:**

This study aimed to analyze health care utilization and direct medical costs of patients with prostate cancer in China.

**Methods:**

Health care service data with a national representative sample of basic medical insurance beneficiaries between 2015 and 2017 were obtained from the China Health Insurance Association database. We conducted descriptive and statistical analyses of health care utilization, annual direct medical costs, and composition based on cancer-related medical records. Health care utilization was measured by the number of hospital visits and the length of stay.

**Results:**

A total of 3,936 patients with prostate cancer and 24,686 cancer-related visits between 2015 and 2017 were identified in the database. The number of annual outpatient and inpatient visits per patient differed significantly from 2015 to 2017. There was no obvious change in length of stay and annual direct medical costs from 2015 to 2017. The number of annual visits per patient (outpatient: 3.0 vs. 4.0, P < 0.01; inpatient: 1.5 vs. 2.0, P < 0.001) and the annual medical direct costs per patient (US$2,300.1 vs. US$3,543.3, P < 0.001) of patients covered by the Urban Rural Resident Basic Medical Insurance (URRBMI) were both lower than those of patients covered by the Urban Employee Basic Medical Insurance (UEBMI), and the median out-of-pocket expense of URRBMI was higher than that of UEBMI (US$926.6 vs. US$594.0, P < 0.001). The annual direct medical costs of patients with prostate cancer in Western regions were significantly lower than those of patients in Eastern and Central regions (East: US$4011.9; Central: US$3458.6; West: US$2115.5) (P < 0.001).

**Conclusions:**

There was an imbalanced distribution of health care utilization among regions in China. The direct medical costs of Chinese patients with prostate cancer remained stable, but the gap in health care utilization and medical costs between two different insurance schemes and among regions still needed to be further addressed.

## Introduction

Prostate cancer is the second most prevalent cancer in males in the world (Bray et al., 2018; Culp et al., 2020). Incidence and mortality rates of prostate cancer are on the decline or have stabilized recently in most developed countries (Culp et al., 2020), but they demonstrate an increasing trend in some developing countries like China (Rongshou et al., 2019; Culp et al., 2020). Prostate cancer is the sixth common malignancy and the tenth leading cause of cancer death in Chinese men, and its 5‐year survival rate (66.4%) in China was much lower than that in the United States (99.5%) (Rongshou et al., 2019).

Prostate cancer is one of the highest increasing cancers in terms of medical costs with a tremendous economic burden (Norlund et al., 2003; Fourcade et al., 2010; Felix et al., 2011; Mariotto et al., 2011; Luengo-Fernandez et al., 2013; Mervin et al., 2017; Restelli et al., 2017; Satoh et al., 2018; Zhuo et al., 2019). Previous studies have shown that metastatic progression contributed to an increase in costs and medical resource use for prostate cancer patients (Penson et al., 2004; Li et al., 2017; Satoh et al., 2018). Being older at diagnosis and higher comorbidity would affect treatment choice of prostate cancer patients and be associated with increased costs (Wilson et al., 2007; Krahn et al., 2010). Studies on the economic burden or costs of prostate cancer were mainly focused on the United States, Europe countries (Sanyal et al., 2013; Smith-Palmer et al., 2019), or other developed countries (Grover et al., 1999; Grover et al., 2000; Krahn et al., 2010; Kitazawa et al., 2015; Mervin et al., 2017; Satoh et al., 2018). Only a few studies described the medical expenditure of Chinese patients with metastatic prostate cancer and presented a high prevalence of bone metastasis among Chinese prostate cancer patients (Qian et al., 2019; Zhuo et al., 2019). However, evidence on health care utilization and medical expenditure focused on prostate cancer has been absent in China. We aimed to analyze the health care utilization and direct medical costs of patients with prostate cancer in China.

## Methods

### Data Source

There are two separate social health insurance schemes in China (Fang et al., 2019): the Urban Rural Resident Basic Medical Insurance (URRBMI), which covers the urban non-employed and self-employed population and the rural population and was formed by merging the Urban Resident Basic Medical Insurance (URBMI) and the New Rural Cooperative Medical Scheme (NRCMS) in 2016 (Pan et al., 2016); the second is the Urban Employee Basic Medical Insurance (UEBMI), which covers the urban employed population and retired people. In 2015, about 97% of the population was covered by one of these basic medical insurance (BMI) schemes (Zhang et al., 2019).

The China Health Insurance Association (CHIRA) employs a two-stage design to obtain a national representative sample of BMI beneficiaries and extracts cross-sectional medical service utilization data annually from the city-level BMI databases. Using probability proportionate to size sampling, the percentage of sampled BMI beneficiaries accounts for 2% population of the centrally administered municipalities and provincial capitals, 5% of the prefecture-level cities, and 10% of the counties (Xia et al., 2015). The details of the CHIRA database could be found in other published studies (Xia et al., 2015; Zong et al., 2016; Huang et al., 2017; Zhang et al., 2019).

This study was based on the CHIRA database between 2015–2017, which contained detailed inpatient and outpatient record information, including patient’s age, sex, diagnoses, medications, and costs of BMI beneficiaries sampled from 82 cities nationwide, representing about 2% of the total population in mainland China (Zhang et al., 2019).

The need for ethics approval was waived by the Ethics Committee of Peking University Health Science Center, Beijing, China (No. IRB00001052-19017), on the basis that the data from the data source in the study, China Health Insurance Association (CHIRA) database, is anonymized and de-identified. No identified or potentially identifiable human information was collected or generated in this study.

### Study Population Identification

**Figure 1:**
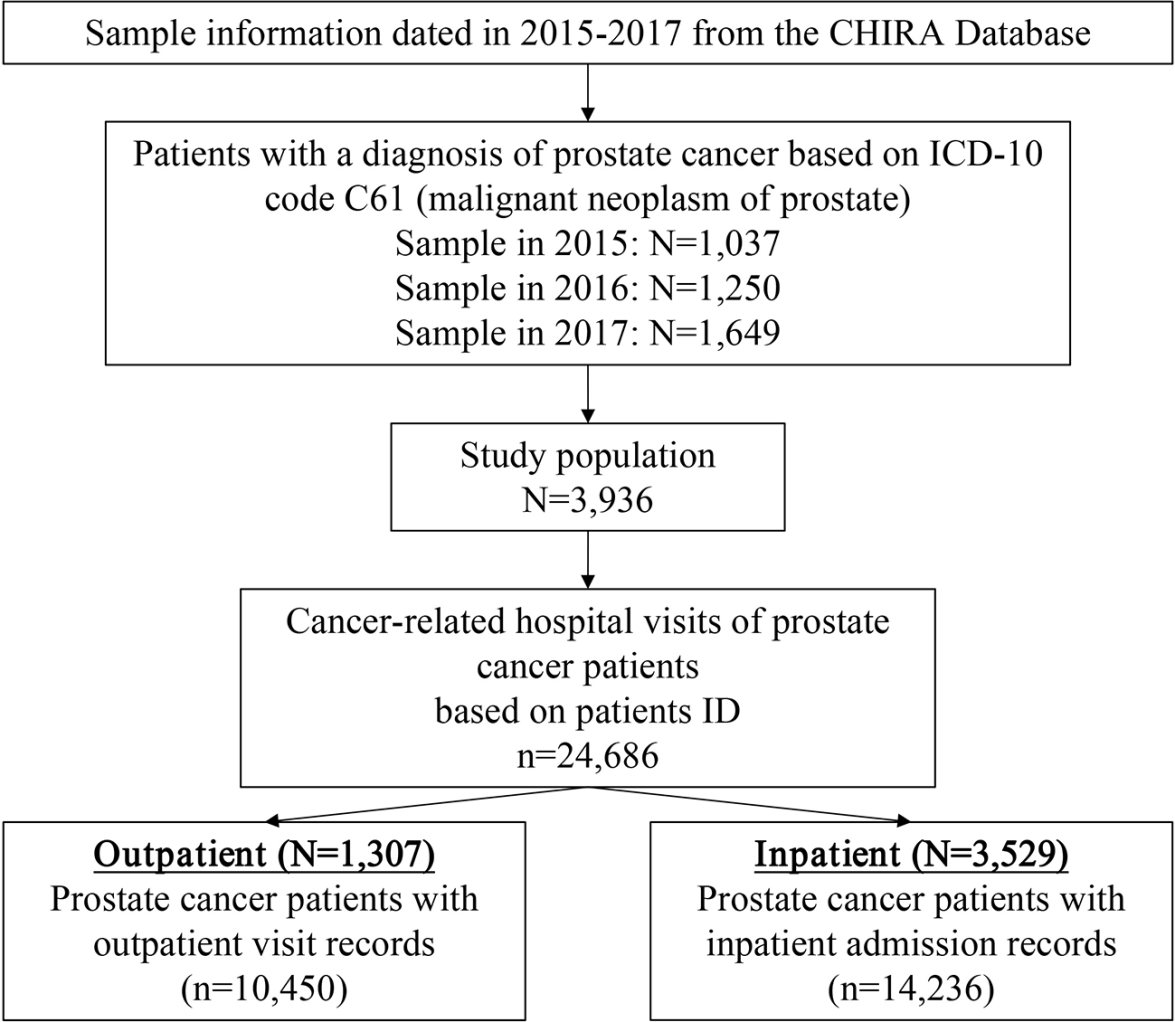
Diagram of sample identification process from the CHIRA database. CHIRA, the China Health Insurance Association; ICD-10, the International Classification of Diseases 10th Revision.

Patients in the sample who had been diagnosed with prostate cancer were identified as individuals with the International Classification of Disease 10th revision (ICD-10) code C61 (malignant neoplasm of prostate). Cancer-related medical records of sample patients were extracted based on patient ID (**Figure 1**).

### Outcomes

In terms of health care utilization, the number of annual outpatient visits per patient was adopted for outpatients; the number of annual inpatient visits per patient and length of stay was adopted for inpatients. We also applied annual direct medical costs per patient and out-of-pocket (OOP) payments, including medications, surgery, examination, nursing care, and other medical management cost. We then calculated the direct medical costs per patient by visit type, the percentages of medication costs per patient, and the percentages of OOP payments per patient. Costs in United States dollars (USD) were calculated using the annual data of the Consumer Price Index in China in 2017 based on the average conversion rate of 6.8 ¥/US$ in 2017 (China, 2018a).

### Statistics Analysis

Numerical variables were described by median value and interquartile range (IQR). To test the differences between several groups, measurements for prostate cancer patients were compared by demographics and clinical variables, using the Mann Whitney U test and Welch’s test for analysis of variance (Welch’s ANOVA). All analyses were conducted in STATA 15, and 2-tailed P <0.05 was considered statistically significant.

## Results

### Characteristics of the Study Sample

A total of 3,936 patients with prostate cancer and 24,686 cancer-related visits between 2015 and 2017 were identified in the database (**Figure 1**). A total of 94.6% of the patients with prostate cancer included in this study were over 60 years old, with an average age of 74.4 years (SD 8.9). Eastern regions accounted for most patients in the sample (45.3%), while Western regions accounted for only 22.4% (**Table 1**).

**Table 1:** Characteristics and health care utilization of patients with prostate cancer.

Characteristics		Sample Characteristics			Health care utilization								
		*No. (%) of outpatient* *(n = 1307)*	*No. (%) of inpatient* *(n = 3529)*	*No. (%) of total patients* *(n = 3936)*	*No. of outpatient visits, median (IQR)^a^*	*P value*	*No. of inpatient hospital admission, median (IQR)*	*P value*	*Length of stay, median (IQR), d*		*P value*	
**Year**												
	*2015*	357 (27.3)	887 (25.1)	1037 (26.4)	5.0 (1.0, 11.0)	<0.01	2.0 (1.0, 4.0)	<0.001	16.0 (8.0, 35.0)		0.9600	
	*2016*	421 (32.2)	1127 (31.9)	1250 (31.8)	4.0 (2.0, 10.0)		2.0 (1.0, 6.0)		18.0 (9.0, 35.0)			
	*2017*	529 (40.5)	1515 (42.9)	1649 (41.9)	4.0 (2.0, 8.0)		2.0 (1.0, 6.0)		18.0 (9.0, 32.0)			
**Age, mean (SD)**^**b**^**, years**		74.1 (9.4)	74.4 (8.7)	74.4 (8.9)	–	–	–	–		–		–	
**Health insurance scheme**													
	*URRBMI*^c^	122 (9.3)	612 (17.3)	632 (16.1)	3.0 (1.0, 6.0)	<0.001	1.5 (1.0, 4.0)	<0.001	14.0 (7.0, 26.0)		<0.001	
	*UEBMI*^d^	1185 (90.7)	2917 (82.7)	3304 (83.9)	4.0 (2.0, 10.0)		2.0 (1.0, 6.0)		18.0 (9.0, 36.0)	
**Region**													
	*Eastern*	979 (74.9)	1453 (41.2)	1782 (45.3)	5.0 (2.0, 11.0)	<0.001	4.0 (1.0, 8.0)	<0.001	18.0 (10.0, 33.0)		<0.001	
	*Central*	149 (11.4)	1241 (35.2)	1272 (32.3)	3.0 (1.0, 7.0)		2.0 (1.0, 5.0)		23.0 (10.0, 49.0)	
	*Western*	179 (13.7)	835 (23.7)	882 (22.4)	2.0 (1.0, 5.0)		1.0 (1.0, 2.0)		12.0 (6.0, 22.9)	
**With other cancer**													
	*No*	1123 (85.9)	3234 (91.6)	3615 (91.8)	4.0 (1.0, 9.0)	<0.001	2.0 (1.0, 5)	<0.001	17.0 (9.0, 32.0)		<0.001	
	*Yes*	184 (14.1)	295 (8.4)	321 (8.2)	7.0 (2.0, 15.0)		4.0 (2.0, 8.0)		29.0 (14.0, 54.0)	

a IQR, interquartile range.

b SD, standard deviation.

c URRBMI, The Urban Rural Resident Basic Medical Insurance.

d UEBMI, The Urban Employee Basic Medical Insurance.

### Health Care Utilization

The number of annual outpatient visits per patient in 2015 was significantly higher than those in 2016 and 2017 (2015: 5.0; 2016: 4.0; 2017: 4.0) (P < 0.01), and the number of annual inpatient visits per patient in 2015 was lower than those in 2016 and 2017 (P < 0.001). There was no obvious change in the length of stay from 2015 to 2017. The number of annual outpatient visits per patient (4.0 vs. 3.0, P < 0.001) and the number of annual inpatient visits (2.0 vs. 1.5, P < 0.001) of UEBMI were both significantly higher than those of URRBMI. In terms of regions, the number of annual outpatient visits of patients (East: 5.0; Central: 3.0; West: 2.0) (P < 0.001) and the number of annual inpatient visits per patient (East: 4.0; Central: 2.0; West: 1.0) (P < 0.001) in the Eastern region were both significantly higher than those in Central and Western regions. For patients covered by the same insurance scheme, the difference in inpatient visits among the three regions existed (P < 0.001) (**Supplementary Table 1**). The numbers of annual visits of patients with other cancer diagnoses and with prostate cancer only were significantly different (P < 0.001). Besides, patients with other cancer had a longer hospitalization compared to patients with prostate cancer only (29.0 vs. 17.0, P < 0.001).

### Direct Medical Costs

The median annual direct medical costs of outpatients and inpatients were US$399.8 and US$3,557.5, respectively (**Table 2**). The median annual medication expenditure per patient was US$1,851.1 and accounted for 62.8% of the total costs. The OOP of patients with prostate cancer was US$622.4, accounting for 21.7% of total costs. From 2015 to 2017, the annual direct medical costs of outpatient per patient decreased (2015: US$508.1; 2016: US$430.3; 2017: US$338.9) (P < 0.001), and no significant change was observed in the total expenditure and OOP. The median annual direct costs of patients covered by UEBMI and URRBMI were significantly different (US$3,543.3 vs. US$2,300.1, P < 0.001). The median annual OOP payment of patients covered by URRBMI was higher than that of patients covered by UEBMI (US$926.6 vs. US$594.0, P < 0.001). The median direct medical costs of patients in Western regions were US$2,115.5, lower than those of patients in Eastern and Central regions (P < 0.001). The median proportion of medication costs per patient in the Eastern region was 70.4%, significantly higher than other regions (P < 0.001). The differences across regions still existed when analyzed separately for patients covered by URRBMI or UEBMI (**Supplementary Table 1**).

**Table 2:** Annual direct medical costs and demographics of patients with prostate cancer in China between 2015 and 2017.

Characteristics		Outpatient		Inpatient		Total									
		(n = 1307)		(n = 3529)		(N = 3936)									
		*Annual direct medical costs of outpatients, meidan (IQR)^a^, USD^b^*	*P value*	*Annual direct medical costs of inpatients, meidan (IQR), USD*	*P value*	*Annual direct medical costs, meidan (IQR), USD*	*P value*	*Annual medication costs, meidan (IQR), USD*	*P value*	*Percentages of medication costs, meidan (IQR), %*	*P value*	*Annual OOP^c^, meidan (IQR), USD*	*P value*	*Percentages of OOP, meidan (IQR), %*	*P value*
**All patients**		399.8 (128.5, 1174.8)	/	3557.5 (1488.9, 7280.9)	/	3306.0 (1315.8, 7027.1)	/	1851.1 (685.1, 4053.3)	/	62.8 (40.0,83.6)	/	622.4 (222.2, 1572.9)	/	21.7 (12.6,33.4)	/
**Year**															
	*2015*	508.1 (131.3, 1694.4)	< 0.001	3214.6 (1265.3, 7246.5)	0.4470	2991.2 (1093.3, 6825.3)	0.6230	1617.7 (614.7, 3895.8)	0.1140	64.2 (41.3,86.1)	< 0.05	563.5 (187.0, 1525.9)	0.1920	21.6 (13.2,33.0)	< 0.001
	*2016*	430.3 (138.3, 1094.2)		3958.3 (1,554.3, 7975.9)		3611.3 (1346., 7641.6)		1987.0 (722.4, 4556.5)		62.9 (40.6,83.2)		620.0 (240.2, 1605.)		19.3 (12.1,29.7)	
	*2017*	338.9 (124.3, 954.9)		3650.4 (1612.2, 7130.3)		3468.3 (1487.4, 6993.8)		1938.6 (741.2, 4054.2)		61.9 (38.0,81.9)		688.9 (238.0, 1662.2)		24.4 (13.0,36.1)	
**Health insurance scheme**															
	*URRBMI*^d^	192.5 (53.4, 517.6)	< 0.001	2359.4 (977.3, 5,141.5)	< 0.001	2300.1 (903.4, 5273.6)	< 0.001	1095.0 (464.2, 2982.9)	< 0.001	54.6 (36.7,76.1)	< 0.001	926.6 (281.4, 2148.9)	< 0.001	41.2 (25.8, 53.8)	< 0.001
	*UEBMI*^e^	429.7 (136.9, 1232.7)		3922.4 (1625.0, 7728.3)		3543.3 (1406.9, 7393.8)		2009.6 (749.9, 4277.2)		64.3 (40.6,85.2)		594.0 (215.8, 1467.0)		20.0 (11.8, 29.7)	
**Region**															
	*Eastern*	515.6 (149.5, 1486.7)	< 0.001	4593.4 (1994.5, 8343.6)	< 0.001	4011.9 (1525.7, 7762.8)	< 0.001	2459.3 (914.4, 4751.4)	< 0.001	70.4 (45.0, 92.8)	< 0.001	676.5 (227.9, 1711.5)	< 0.001	20.1 (11.0, 33.5)	< 0.001
	*Central*	270.3 (73.2, 619.3)		3515.8 (1464.7, 7783.2)		3458.6 (1381.5, 7709.3)		1800.1 (673.7, 4213.2)		57.2 (36.0, 75.4)		898.6 (371.0, 1948.8)		26.7 (19.4, 36.1)	
	*Western*	177.5 (66.3, 416.7)		2202.2 (1111.1, 4795.)		2115.5 (1001.5, 4732.5)		1070.4 (502.2, 2462.8)		59.0 (37.5, 78.8)		313.0 (106.9, 761.5)		15.1 (10.0, 26.7)	
**With other cancer**															
	*No*	385.1 (124.1, 1125.6)	< 0.05	3414.7 (1427.8, 6961.5)	< 0.001	3112.9 (1232.7, 6673.7)	< 0.001	1715.3 (632.2, 3845.3)	< 0.001	62.8 (39.5, 83.8)	0.9113	586.2 (207.6, 1487.7)	< 0.001	21.5 (12.5, 33.2)	< 0.01
	*Yes*	494.4 (171.6, 1265.1)		6127.8 (2721.5, 11366.2)		6206.1 (2970.8, 11363.5)		3546.4 (1788.1, 6146.2)		62.2 (43.8, 81.9)		1226.1 (492.0, 3160.3)		24.7 (15.4, 35.7)	

a IQR, interquartile range.

b USD, United States dollar.

c OOP, out-of-pocket.

d URRBMI, The Urban Rural Resident Basic Medical Insurance.

e UEBMI, The Urban Employee Basic Medical Insurance.

## Discussion

Our study revealed that the number of cancer-related outpatient visits decreased from 2015 to 2017 and the number of cancer-related inpatient visits increased in the 3 years. The median annual direct medical costs of patients with prostate cancer did not increase from 2015 to 2017. However, there were notable differences among regions and between two health insurance schemes.

From 2015 to 2017, the median annual direct medical costs observed in this study stayed stable, lower than those observed in other countries (Alemayehu et al., 2010; Satoh et al., 2018). Besides, the OOP of patients with prostate cancer in 2015, 2016, and 2017 accounted for 44.4%, 35.6%, and 34.5% of personal disposable income per capita (China, 2018a) in the given year, respectively. However, the trend of medical costs in our study differed from prior studies in other countries, which showed that the direct medical costs of prostate cancer and the personal medical economy burden of patients with prostate cancer had been increasing (Crawford et al., 2010; Mariotto et al., 2011; Zhuo et al., 2019). This disparity may be due to the effective control of medication costs. The Chinese government started comprehensive health system reform in 2009 and issued a series of policies (Liu et al., 2017). The government established maximum retail prices for specific products in 2012 and then abolished price regulation for most price-regulated medications, including all antineoplastic medications in 2015, which made expenses of the price-regulated antineoplastic medications drop significantly (Guan et al., 2019). In addition, the Chinese government gradually implemented zero make-up policy on drug prices, which controlled medication costs and relief the personal medical economy burden of Chinese residents (Liu et al., 2019; Meng et al., 2019).

Besides, the OOP payments of patients covered by two insurance schemes significantly differed. Patients covered by URRBMI had lower health care utilization and direct medical costs than those covered by UEBMI but paid higher OOP, the same as the prior studies on medical expenses of other diseases (Feng et al., 2013; Yong et al., 2018; Yuan et al., 2019), which is related to the discrepant co-payment level of the two health insurance schemes. The major funding sources for UEBMI are payroll taxes and the major funding sources for URRBMI are government subsidies so that the insurance premiums collected from UEBMI are much higher. Hence, UEBMI offers a wider health care service coverage and a higher proportion of insurance reimbursement than URRBMI (Meng et al., 2015). Research has shown that fragmentation in social health insurance schemes would be an important factor for inequitable access to health care and financial protection for people covered by different insurance schemes and would affect the level of disease control (Feng et al., 2014; Meng et al., 2015). Consolidating the schemes and their co-payment levels through more government funding would reduce OOP expenses for patients covered by URRBMI (Fang et al., 2019) and improve the equity of health care and financial protection.

There were significant differences in health care utilization and costs among regions. The number of annual outpatient and inpatient visits, the length of stay, annual direct medical costs, and OOP of patients in Western regions were much lower than those in Eastern and Central regions, while the number of hospital visits and the proportion of medication costs per capita in Eastern regions were higher than that in other regions. Regional imbalance in economic and health care development may contribute to this phenomenon. The economically developed regions have a higher level of health resources and services (Zhang et al., 2015; Zhang et al., 2017; China, 2018b) and higher personal disposable income per capita (China, 2018a) than underdeveloped regions. Therefore, patients in developed regions always have a higher demand for medical services (Okunad and Murthy, 2002; Chandra and Skinner, 2012; Leth-Petersen and Skipper, 2014; You and Okunade, 2017) and a higher expectation of disease control and prognosis, and they would be more willing to visit hospitals and choose a treatment with a higher price. The life expectancy of residents in developed regions is obviously superior to that in underdeveloped regions (Zhou et al., 2016). To balance the level of health care services and utilization, a further increase in health resources and funding should be more focused on underdeveloped regions.

This study had several limitations. First, the CHIRA database excluded people who were not covered by BMI, which might affect the representativeness of the study sample. However, its impact on the results should be limited because the people uncovered by BMI accounted for very few of the whole population in China. Second, the CHIRA database obtained a national representative sample from the city-level BMI databases separately each year. It was unable to track patient data for more than one year and failed to conduct a longitudinal analysis. Moreover, we failed to capture the whole health care utilization and costs if the patients were diagnosed with prostate cancer firstly during the sampling year, which might make our results biased. Third, the CHIRA database had no further diagnostic information, and we were unable to identify the cancer progression of sample patients. Although the health care utilization and costs of patients with prostate cancer were usually different across cancer levels, we could not carry out a stratified analysis of patients with different levels. Fourth, patients that visited hospitals in their non-registered locations in the sample needed to pay all direct medical costs out of pocket, which could affect the results in our study. In addition, we could not conclude whether OOP would lead to increasing family financial burden because family income information was not recorded in the CHIRA database. Despite these limitations, the CHIRA database can be considered an “extensive health insurance database” compared to other data sources in China, and this unique and representative national health insurance database would be important to advance Chinese population-based research for the integration of real-world evidence into clinical practice and local policymaking.

## Conclusions

This study found that there was an imbalanced distribution of health care utilization among regions in China, with fewer health care resources consumed in Western regions. The direct medical costs of Chinese patients with prostate cancer remained stable and were lower than those in other countries. However, the gap in health care utilization and costs between two different insurance schemes and among regions still need to be addressed further.

## Data Availability Statement

The datasets generated for this study are available on request to the corresponding author.

## Ethics Statement

The need for ethics approval was waived by the Ethics Committee of Peking University Health Science Center, Beijing, China (No. IRB00001052-19017), on the basis that the data from the data source in the study, China Health Insurance Association (CHIRA) database, is anonymized and de-identified. No identified or potentially identifiable human information was collected or generated in this study.

## Author Contributions

The contributions made by the individual authors are as follows. LS and XG conceptualized and designed the study. CH, ZL, and HW contributed to the data analysis. LB and XG conducted the final analyses. LB, XG, HW, and LS drafted the initial manuscript. All authors contributed to the article and approved the submitted version.

## Conflict of Interest

The authors declare that the research was conducted in the absence of any commercial or financial relationships that could be construed as a potential conflict of interest.
