# Supplementary material for: Health Care Utilization and Costs of Patients With Prostate Cancer in China Based on National Health Insurance Database From 2015 to 2017
Source: Front Pharmacol. 2020 Jun 10;11:719. doi: 10.3389/fphar.2020.00719 (PMC7299164; doi:10.3389/fphar.2020.00719)
Supplement: Supplementary file 1 [file Table_1.docx]

**Supplementary Table 1 The difference among regions of prostate cancer patients covered by URRBMI and UEBMI in China between 2015 and 2017**

|  | | **URRBMI^a^** | | | |  | **UEBMI^b^** | | | |
| --- | --- | --- | --- | --- | --- | --- | --- | --- | --- | --- |
|  |  | **(n = 632)** | | | |  | **(n = 3304)** | | | |
|  |  | ***Eastern*** | ***Central*** | ***Western*** | ***P value*** |  | ***Eastern*** | ***Central*** | ***Western*** | ***P value*** |
| **Health care utilization** | |  |  |  |  |  |  |  |  |  |
|  | *No. of outpatient visits, median (IQR)^c^* | 4.0 | 1.0 | 2.5 | 0.208 |  | 5.0 | 3.0 | 2.0 | <0.001 |
|  |  | (1.0, 7.0) | (1.0, 6.0) | (1.0, 4.0) |  |  | (2.0, 11.0) | (1.0, 7.0) | (1.0, 5.0) |  |
|  | *No. of inpatient hospital admission, median (IQR)* | 4.0 | 2.0 | 1.0 | <0.001 |  | 3.0 | 2.0 | 1.0 | <0.001 |
|  |  | (1.0, 8.0) | (1.0, 3.0) | (1.0,1.0) |  |  | (1.0, 8.0) | (1.0, 5.0) | (1.0, 3.0) |  |
|  | *No. of outpatient visits, median (IQR)* | 15.0 | 16.5 | 10.0 | <0.05 |  | 18.0 | 24.0 | 13.0 | <0.001 |
|  |  | (9.5, 25.5) | (8.0, 33.0) | (5.0, 17.0) |  |  | (10.0, 34.0) | (11.0, 51.0) | (7.0, 24.2) |  |
| **Annual direct medical costs and demographics** | |  |  |  |  |  |  |  |  |  |
|  | *Annual direct medical costs of outpatients, meidan (IQR), USD^d^* | 205.9 | 43.3 | 199.3 | 0.07 |  | 564.4 | 317.6 | 177.5 | <0.001 |
|  |  | (58.9, 583.8) | (17.8, 386.7) | (91.6, 627.0) |  |  | (168.1, 1605.8) | (81.1, 636.0) | (63.3, 399.6) |  |
|  | *Annual direct medical costs of inpatients, meidan (IQR), USD* | 3775.9 | 2349.0 | 1318.6 | <0.001 |  | 4744.4 | 3962.7 | 2544.3 | <0.001 |
|  |  | (1719.5, 6830.1) | (866.4, 5011.4) | (600.1, 2729.6) |  |  | (2055.1, 8615.5) | (1564.9, 8467.6) | (1337.5, 5479.4) |  |
|  | *Annual direct medical costs, meidan (IQR), USD* | 3734.7 | 2330.1 | 1318.6 | <0.001 |  | 4066.4 | 3935.5 | 2409.1 | <0.001 |
|  |  | (1547.3, 6691.1) | (837.2, 4963.1) | (593.3, 2729.6) |  |  | (1510.7, 7995.9) | (1524.5,8316.6) | (1157.5,5341.5) |  |
|  | *Annual medication costs, meidan (IQR), USD* | 2263.7 | 910.8 | 652.5 | <0.001 |  | 2474.1 | 2116.1 | 1258.8 | <0.001 |
|  |  | (932.3, 4532.8) | (396.0, 2131.2) | (269.1, 1333.1) |  |  | (913.0, 4814.4) | (808.2, 4673.3) | (558.5, 2869.8) |  |
|  | *Percentages of medication costs, meidan (IQR), %* | 67.9 | 49.0 | 49.1 | <0.001 |  | 70.7 | 58.6 | 62.0 | <0.001 |
|  |  | (47.9, 84.7) | (28.5, 68.7) | (33.5, 67.2) |  |  | (44.6, 93.2) | (37.4, 76.2) | (39.7, 80.6) |  |
|  | *Annual OOP^e^, meidan (IQR), USD* | 1612.7 | 955.5 | 241.2 | <0.001 |  | 588.7 | 888.6 | 333.4 | <0.001 |
|  |  | (674.5, 3049.7) | (385.1, 1998.7) | (75.0, 756.4) |  |  | (207.8, 1479.7) | (369.3, 1938.7) | (111.8, 763.3) |  |
|  | *Percentages of OOP, meidan (IQR), %* | 49.4 | 44.8 | 16.8 | <0.001 |  | 18.0 | 24.6 | 14.8 | <0.001 |
|  |  | (38.9, 60.1) | (32.6, 54.0) | (10.1, 33.7) |  |  | (10.0, 27.9) | (18.3, 31.9) | (10.0, 25.6) |  |

^a^ URRBMI, The Urban Rural Resident Basic Medical Insurance.

^b^ UEBMI, The Urban Employee Basic Medical Insurance.

^c^ IQR, interquartile range.

^d^ USD, United States dollar.

^e^ OOP, out-of-pocket.
